# Machine Learning Analyses Revealed Distinct Arterial Pulse Variability According to Side Effects of Pfizer-BioNTech COVID-19 Vaccine (BNT162b2)

**DOI:** 10.3390/jcm11206119

**Published:** 2022-10-17

**Authors:** Chun-Chao Chen, Che-Kai Chang, Chun-Chih Chiu, Tsung-Yeh Yang, Wen-Rui Hao, Cheng-Hsin Lin, Yu-Ann Fang, William Jian, Min-Huei Hsu, Tsung-Lin Yang, Ju-Chi Liu, Hsin Hsiu

**Affiliations:** 1 Division of Cardiology, Department of Internal Medicine, Shuang Ho Hospital, Taipei Medical University, New Taipei City 23561, Taiwan; 2 Taipei Heart Institute, Taipei Medical University, Taipei 110, Taiwan; 3 Division of Cardiology, Department of Internal Medicine, School of Medicine, College of Medicine, Taipei Medical University, Taipei 110, Taiwan; 4 Graduate Institute of Medical Sciences, College of Medicine, Taipei Medical University, Taipei 110, Taiwan; 5 Graduate Institute of Biomedical Engineering, National Taiwan University of Science and Technology, No. 43, Section 4, Keelung Road, Taipei 10607, Taiwan; 6 Division of Cardiovascular Surgery, Department of Surgery, Shuang Ho Hospital, Taipei Medical University, New Taipei City 235, Taiwan; 7 Division of Cardiovascular Surgery, Department of Surgery, School of Medicine, College of Medicine, Taipei Medical University, Taipei 110, Taiwan; 8 Department of Emergency, University Hospitals Cleveland Medical Center, Cleveland, OH 44106, USA; 9 Graduate Institute of Data Science, College of Management, Taipei Medical University, Taipei 110, Taiwan; 10 Department of Neurosurgery, Shuang Ho Hospital, Taipei Medical University, New Taipei City 235, Taiwan; 11 Division of Cardiology, Department of Internal Medicine and Cardiovascular Research Center, Taipei Medical University Hospital, Taipei 110, Taiwan

**Keywords:** COVID-19 vaccine, side effects, pulse, spectral analysis, machine learning, cardiovascular variability

## Abstract

Various adverse events and complications have been attributed to COVID-19 (coronavirus disease 2019) vaccinations, which can affect the cardiovascular system, with conditions such as myocarditis, thrombosis, and ischemia. The aim of this study was to combine noninvasive pulse measurements and frequency domain analysis to determine if the Pfizer-BioNTech COVID-19 vaccine (BNT162b2) vaccination and its accompanying cardiovascular side effects will induce changes in arterial pulse transmission and waveform. Radial blood pressure waveform and photoplethysmography signals were measured noninvasively for 1 min in 112 subjects who visited Shuang-Ho Hospital for a BNT162b2 vaccination. Based on side effects, each subject was assigned to Group N (no side effects), Group CV (cardiac or vascular side effects), Group C (cardiac side effects only), or Group V (vascular side effects only). Two classification methods were used: (1) machine-learning (ML) analysis using 40 harmonic pulse indices (amplitude proportions, phase angles, and their variability indices) as features, and (2) a pulse-variability score analysis developed in the present study. Significant effects on the pulse harmonic indices were noted in Group V following vaccination. ML and pulse-variability score analyses provided acceptable AUCs (0.67 and 0.80, respectively) and hence can aid discriminations among subjects with cardiovascular side effects. When excluding ambiguous data points, the AUC of the score analysis further improved to 0.94 (with an adopted proportion of around 64.1%) for vascular side effects. The present findings may help to facilitate a time-saving and easy-to-use method for detecting changes in the vascular properties associated with the cardiovascular side effects following BNT162b2 vaccination.

## 1. Introduction

The cardiovascular system may have a bidirectional relationship with COVID-19 (coronavirus disease 2019) infections and may be severely impaired by COVID-19 infection and its secondary consequences [1,2]. Cardiovascular complications are a significant risk factor for COVID-19-associated mortality [3,4]. Patients with preexisting comorbidities such as hypertension, diabetes mellitus, and cardiovascular diseases (CVDs) have been found to be more susceptible to COVID-19 infection and its complications [5].

COVID-19 infection has been suggested to have pathogenic implications for the cardiovascular system and has been found to be associated with the worsening of underlying CVD [6,7]. Myocardial injury significantly increases the amount of serum cardiac troponin in patients with COVID-19. COVID-19 may accelerate cardiac aging, which leads to pulmonary vascular endothelialitis, microangiopathy, diffuse thrombosis, and myocarditis [3]. The protein angiotensin-converting enzyme 2 (ACE2), which is an important cardiovascular regulator, can be involved in the mechanisms of cardiovascular damage [5,8]. Markers of coagulation and inflammatory cytokines have been found to be elevated in patients with COVID-19 [7,9,10].

Elderly patients have the greatest risk of severe COVID-19 and its cardiovascular complications, and so diagnostics and monitoring of severe and critically ill patients constitute the main challenges [3]. Technologies utilizing artificial intelligence (AI) and mobile-health devices may offer new strategies for addressing COVID-19. AI has already been used to predict the spread of the virus, and in the early detection, monitoring, social distancing, and training of healthcare workers during the COVID-19 pandemic. Mobile health apps may improve user-friendliness and therefore strongly support pandemic prevention and control [11].

Various adverse events and complications that reportedly originate from COVID-19 vaccinations can affect the nervous and cardiovascular systems. Some may even be life-threatening, such as acute myocardial infarction, pulmonary embolism, and stroke [12]. Myocarditis, tachycardia, and impaired left ventricular ejection fraction have been noted after receiving the Pfizer-BioNTech COVID-19 vaccine (BNT162b2) vaccination [12]. Monitoring the physiological signs within 7 days of mRNA vaccination is recommended by the Centers for Disease Control and Prevention. Commonly used tools include cardiac magnetic resonance imaging (for which patients need to go to the hospital), ECG signs (for which expert justification is necessary), and inflammation markers (e.g., C-reactive protein and troponin, which requires drawing blood) [13].

Arterial pulses generated by the heartbeat transmit along the artery and propel the blood into the tissues in the microcirculation. Changes in heartbeat, vascular elasticity, or blood supply perfusion conditions may change the coupling relationships between the heart, artery, and vascular beds, and hence change the pulse waveform. The measurement and analysis of pulse waveforms could therefore aid in circulatory system monitoring. Pulse measurement also has application advantages, such as being noninvasive, fast, user-friendly, and objective. Previous studies have analyzed pulse waveforms in the time domain [14,15,16,17] and frequency domain to capture the information buried in the waveform changes induced by CVDs, such as hypertension, stroke, coronary artery disease, vascular aging, and metabolic syndrome, or applying various types of simulation [18,19,20,21,22].

The present study aimed to combine noninvasive pulse measurements and frequency domain analyses to determine if the BNT162b2 vaccination and its accompanying cardiovascular side effects induce changes in pulse waveforms. Since the subjects with cardiovascular side effects constituted only a small proportion of the sample, we developed a scoring system to provide an alternative way to classify subjects. Another aim was to determine if using machine-learning (ML) analysis and the self-developed pulse variability scoring system could help to discriminate vaccinated subjects with cardiovascular side effects. The analysis of pulse waveforms (which helps to monitor the changes induced by vascular elasticity and pulse transmission conditions) in subjects who received vaccination for COVID-19 may offer relevant information and facilitate a noninvasive and easy-to-use method of evaluating its possible impact on the cardiovascular system.

## 2. Methods

### 2.1. Measurement

**Figure 1:**
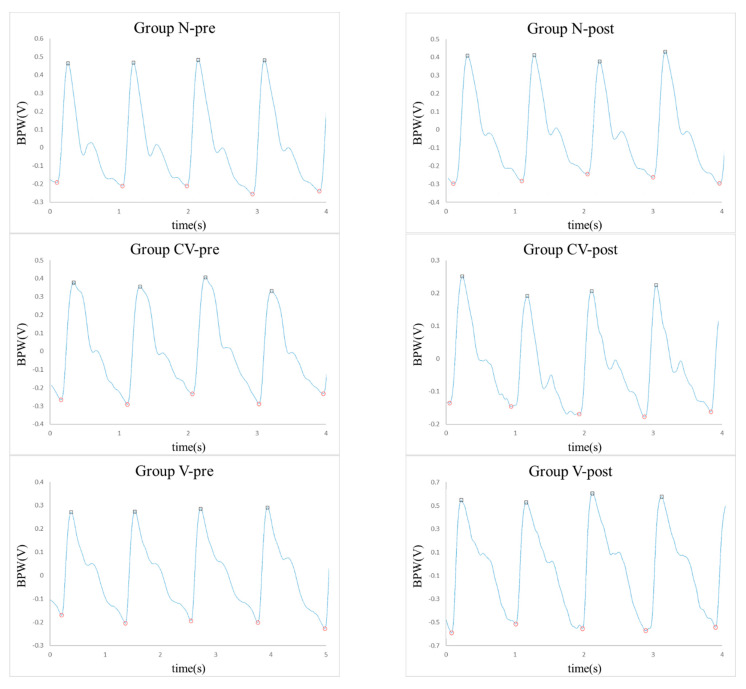

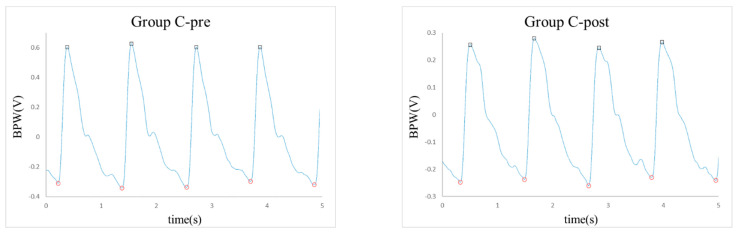
Typical BPWs measured (in arbitrary units).

Details of the present experimental setup and the signal processing methods are available elsewhere [20,21,22,23] and in the Supplemental Materials. Blood pressure waveform (BPW) signals of the subjects were measured noninvasively (typical waveforms are shown in Figure 1).

The subjects were recruited by Shuang-Ho Hospital. Informed consent was obtained from the study participants or their legally designated person (approved by the Review Board of Taipei Medical University; TMU-JIRB N202112027). All experiments were performed in accordance with the relevant guidelines and regulations. The demographic assessments, BPW and photoplethysmography (PPG) measurements, laboratory examinations (urine and blood), 12-lead ECG, and chest X-rays were performed on the subjects before (M0) and 7 days (±3 days) after (M1) BNT162b2 vaccination. Side effects from the vaccines were assessed 7 days (±3 days) after vaccination (schedule and details are listed in Tables S1–S5 in Supplemental Materials).

Measurements were performed on 112 volunteers; details of the subjects are listed in Table 1. The subjects were divided into the following groups: Group N, no side effects; Group CV, with cardiac or vascular side effects; Group C, with cardiac side effects only; and Group V, with vascular side effects only. Cardiac and vascular side effects associated with COVID-19 vaccination are defined in Table 2. A subject was assigned as “with side effect” if they had any one of the listed items.

### 2.2. Analysis

The coefficient of variation of the heart rate (HR_CV) was calculated from the intervals of two neighboring BPW foots to evaluate changes in heart rate (HR) variability. Frequency domain analysis was performed to derive the following 40 BPW harmonic indices for *n* = 1–10: amplitude proportion (*C_n_*), coefficient of variation of *C_n_* (*CV_n_*), phase angle (*P_n_*), and standard deviation of *P_n_* (*P_n_*_*SD*).

Two classification methods were used to discriminate between groups: (1) ML analysis and (2) the self-developed pulse-variability scoring system. For ML analysis (details are provided in the Supplemental Materials), eight supervised methods were used for the binary classification of the data, which were support vector machine (SVM), multilayer perceptron (MLP), Gaussian Naïve Bayes (GNB), decision tree (DT), random forest (RF), logistic regression (LR), linear discriminant analysis (LDA), and K-nearest neighbor (KNN). The features were the 40 indices for each pulse (*n* = 1–10): *C_n_*, *CV_n_*, *P_n_*, and *P_n_*_*SD*. Threefold cross-validation was used in the model training process.

Pulse indices were further used to understand discrimination ability by utilizing the following self-developed pulse-variability scoring system:

▪ We first selected *CV*_2_ and *P*_1__*SD*–*P*_5__*SD* of BPW, since there were significant differences among these.
▪ Using average value of each selected index, the pulse-variability score for the data point of each subject was calculated as:
  [(*CV*_2_M1_)×(*P*_1__*SD*_M1_)×…×(*P*_5__*SD*_M1_)]/[(*CV*_2_M0_)×(*P*_1__*SD*_M0_)×…×(*P*_5__*SD*_M0_)].
▪ We then set threshold levels to study the discrimination ability of the scoring system.

## 3. Result

**Figure 2:**
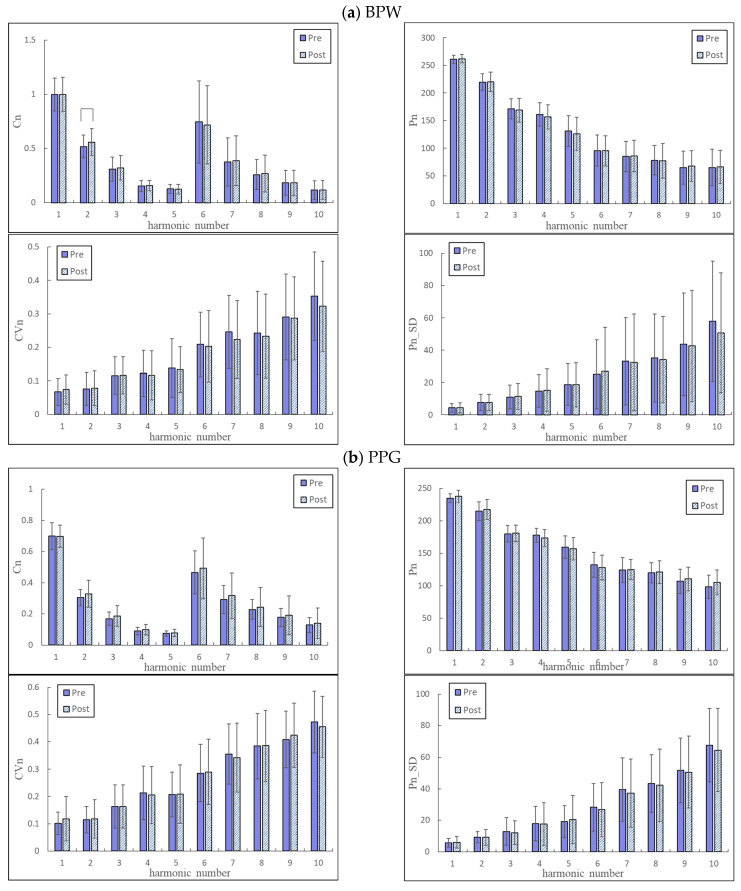
Comparisons of pulse harmonic indices between before (Pre) and after (Post) vaccination (*n* = 75): *C_n_*, *CV_n_*, *P_n_*, and *P_n_*_*SD*. Data are mean and standard deviation values. *C*_5_–*C*_10_ values have been multiplied by 10 to make the differences clearer. “︹” indicates *p* < 0.05. (**a**) BPW; (**b**) PPG. There was only a significant change in *C*_2_ of BPW.

The general characteristics of the participants are listed in Table 1. Figure 2 compares the harmonic indices of the pulse signals between before and after vaccination. Among the BPW and PPG indices, there was only a significant change in *C*_2_ of BPW.

**Figure 3:**
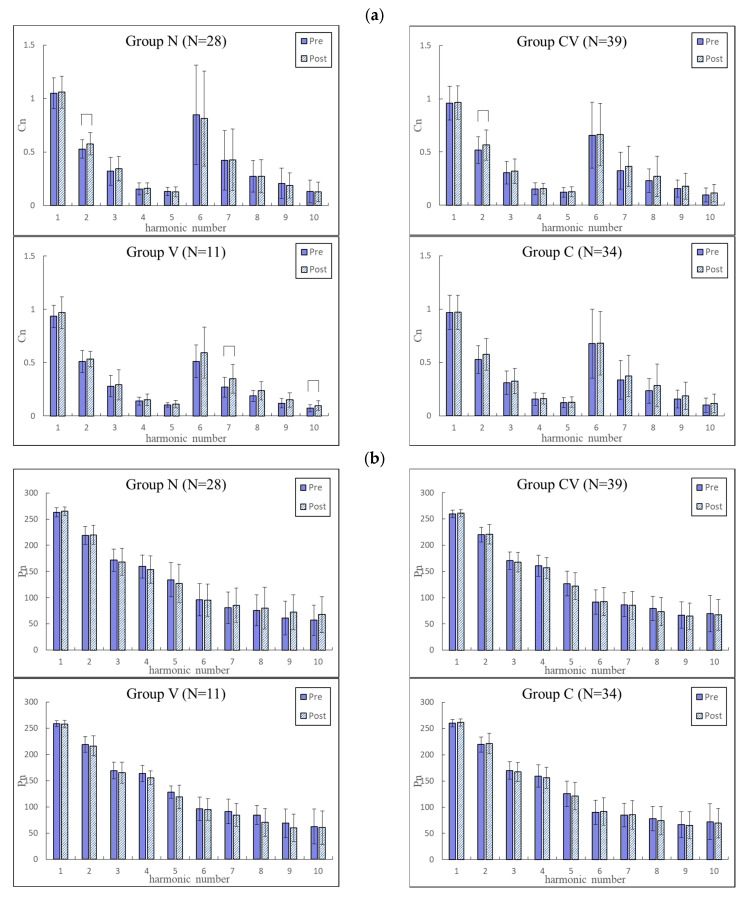

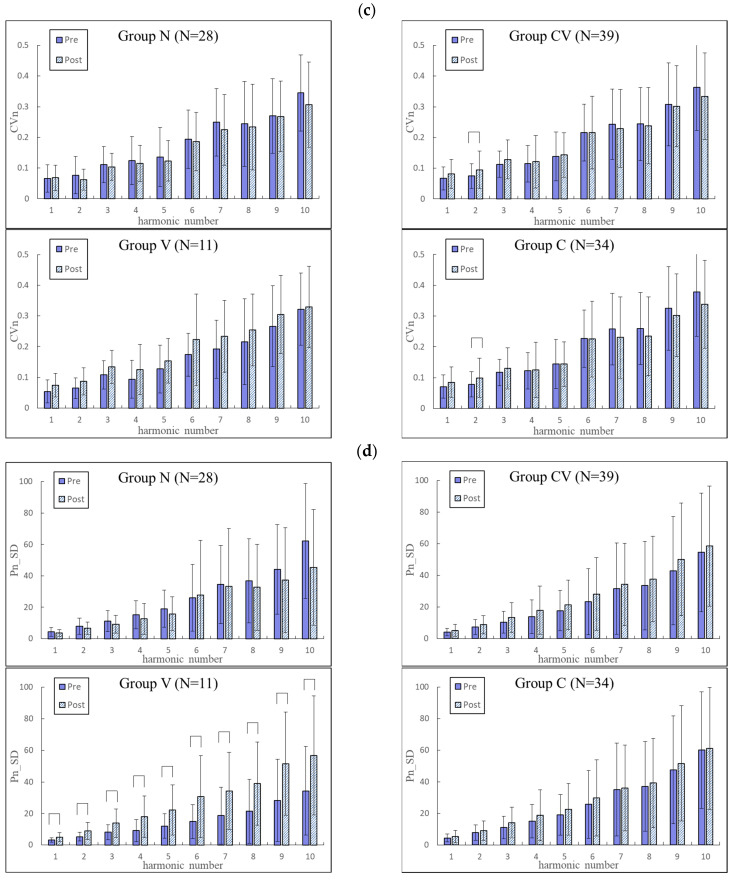
Comparisons of BPW harmonic indices between before and after vaccination in Groups N (*n* = 28), CV (*n* = 39), V (*n* = 11), and C (*n* = 34). Data are mean and standard deviation values. *C*_5_–*C*_10_ values have been multiplied by 10 to make the differences clearer. “︹” indicates *p* < 0.05. (**a**) For *C_n_*, there was no significant change in Group C. In Groups N and CV, there were only significant differences in *C*_2_. There were more prominent changes in Group V, especially in higher frequency components (*C*_6_–*C*_10_; significant in *C*_7_ and *C*_10_). (**b**) There were no significant changes in *P_n_*. (**c**) In Group V, *CV_n_* were all increased (non-significantly). (**d**) In Group V, *P_n_*_*SD* were all significantly increased. There were no significant changes in *CV_n_* and *P_n_*_*SD* in the other groups.

**Figure 4:**
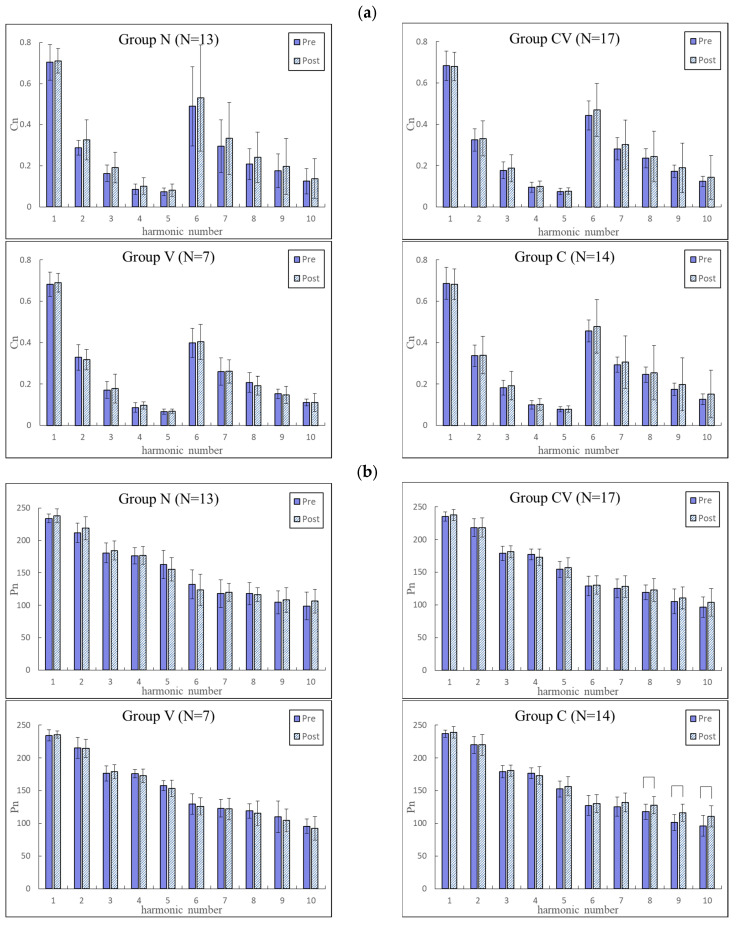

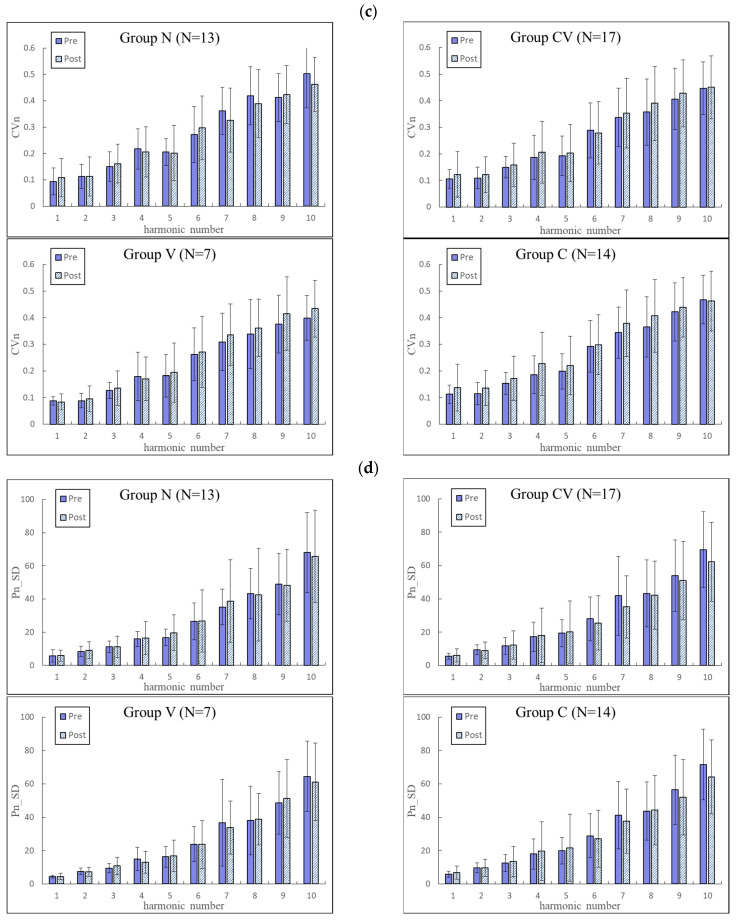
Comparisons of PPG harmonic indices between before and after vaccination in Groups N (*n* = 13), CV (*n* = 17), V (*n* = 7), and C (*n* = 14). (**a**) *C_n_*; (**b**) *P_n_*; (**c**) *CV_n_*; (**d**) *P_n_*_*SD*. Data are mean and standard deviation values. *C*_5_–*C*_10_ values have been multiplied by 10 to make the differences clearer. “︹” indicates *p* < 0.05. The changes in PPG indices were less prominent than those in BPW indices, with only significant changes in *P*_8_–*P*_10_ of PPG.

Figure 3 and Figure 4 compare changes in the pulse indices among the four groups. The BPW indices changed more prominently than did the PPG indices, and the subsequent analysis therefore focused on BPW signals. Among the four groups, Group V had the most prominent changes, especially in the *P_n_*_*SD* indices (all significantly increased).

**Figure 5:**
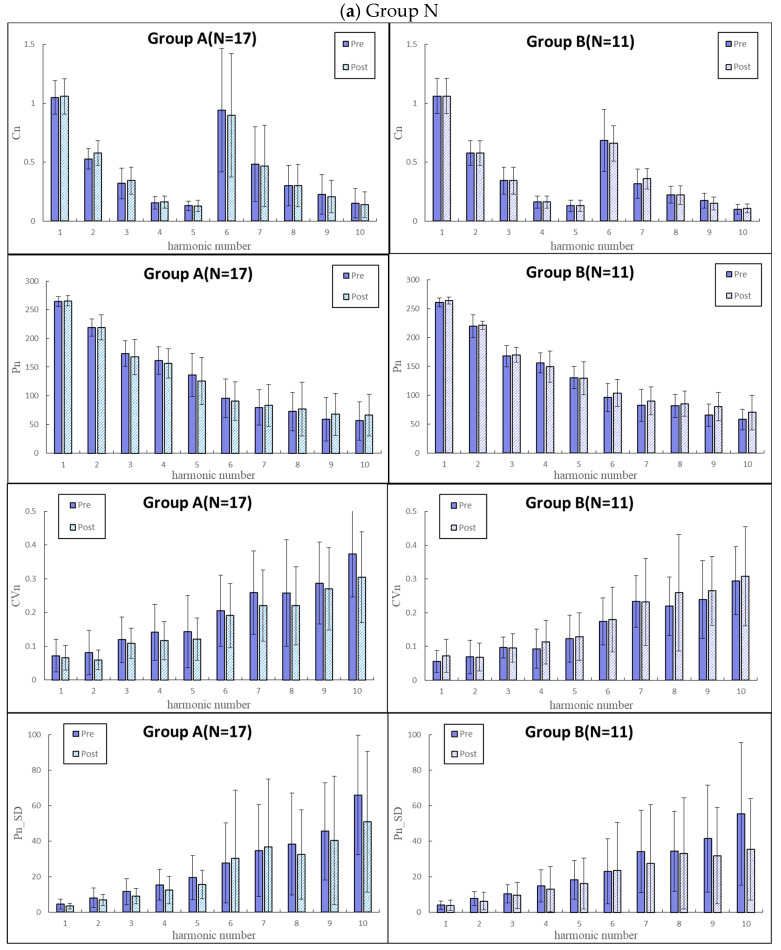

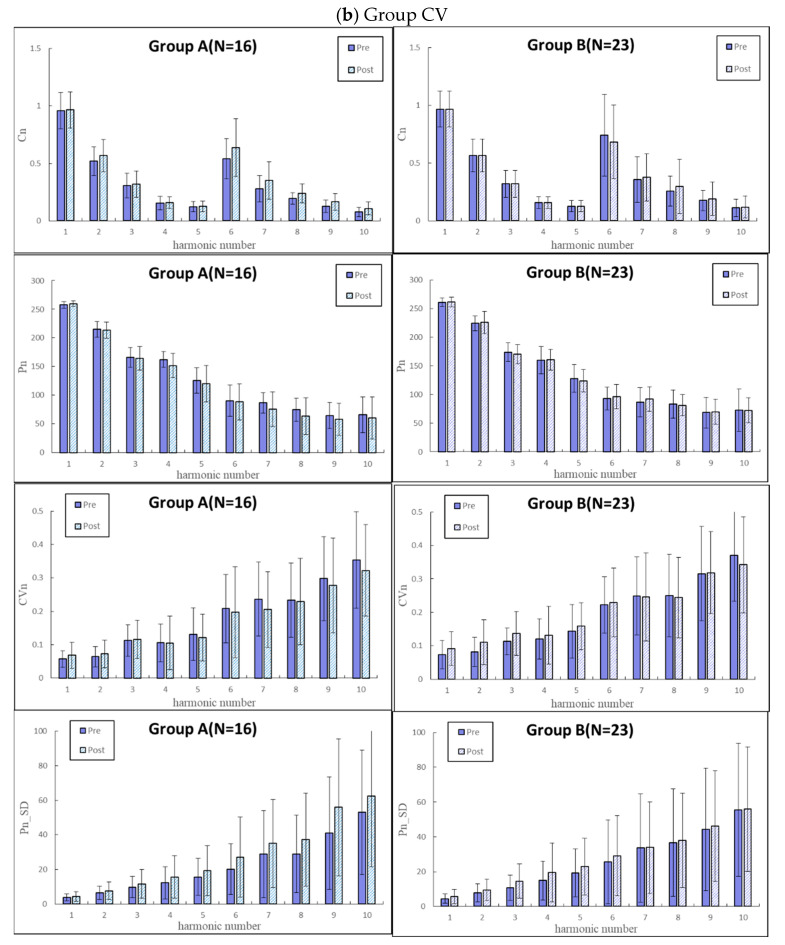

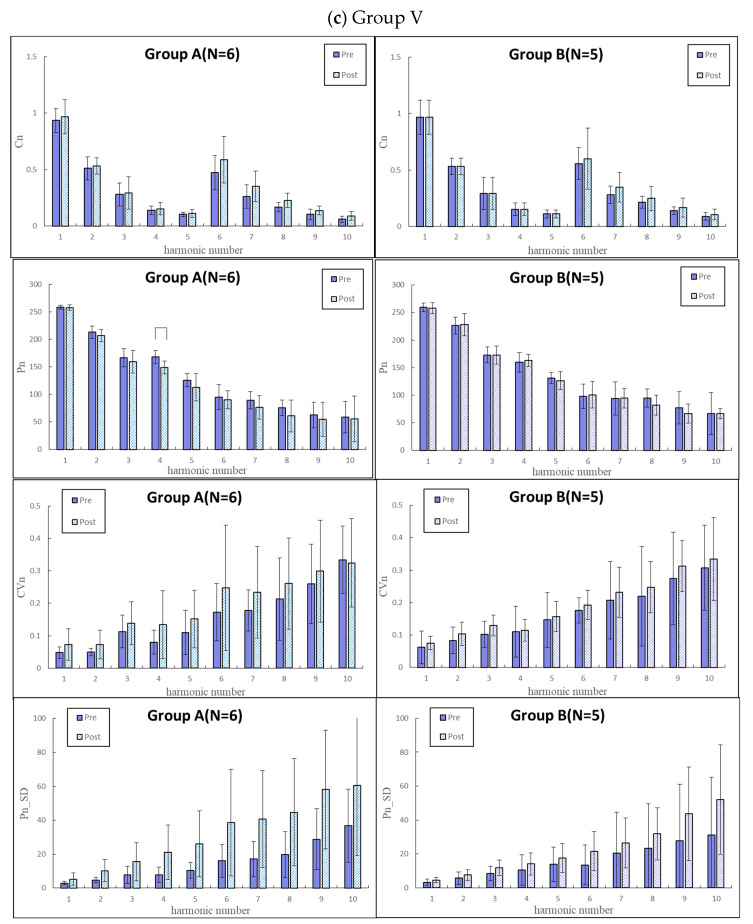

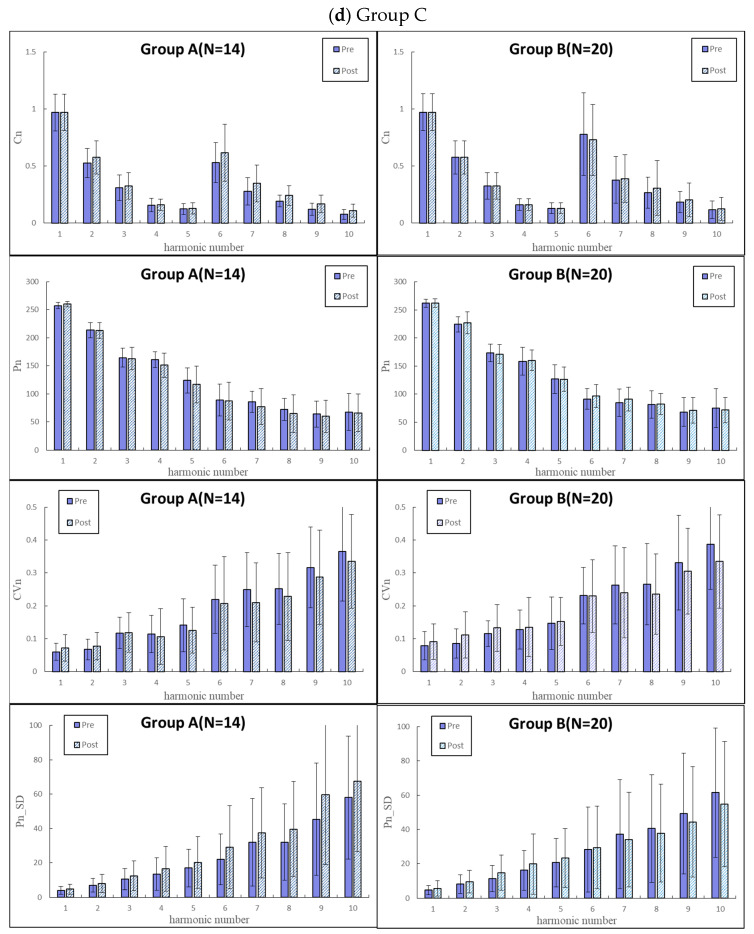
Comparisons of BPW harmonic indices between before and after vaccination in subgroups A (left; with at least one in hypertension, hyperlipidemia, and hyperglycemia) and B (right; without any one of hypertension, hyperlipidemia, and hyperglycemia). (**a**) Group N (*n* = 28); (**b**) Group CV (*n* = 39); (**c**) Group V (*n* = 11); (**d**) Group C (*n* = 34). Data are mean and standard deviation values. *C*_5_–*C*_10_ values have been multiplied by 10 to make the differences clearer. “︹” indicates *p* < 0.05.

For the BPW pulse indices in the four groups in Figure 3, we further divided them into two subgroups (comparison results are shown in Figure 5): subGroups A (with at least one of hypertension, hyperlipidemia, and hyperglycemia) and B (without any one of hypertension, hyperlipidemia, and hyperglycemia). Changes in some BPW indices were more prominent in Group A than in Group B; the most prominent differences were in the *P_n_*_*SD* indices in Groups V. However, there were only few significant changes between pre- and post-vaccination values of the BPW indices.

**Figure 6:**
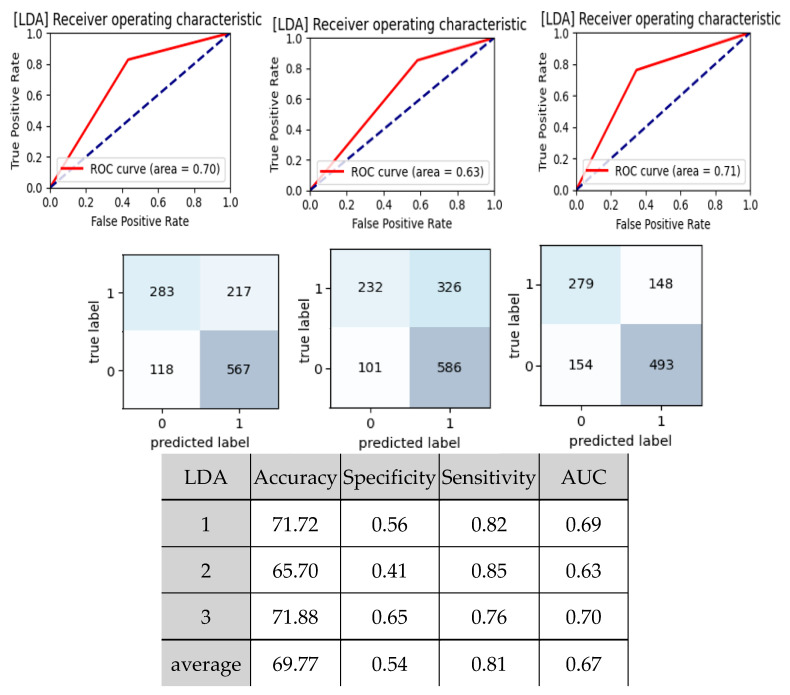
Results of ML analysis (LDA) of BPW indices for discrimination between Groups N and CV using pulse indices as features. “1” denotes cardiovascular side effects; “0” denotes no side effects.

Figure 6 and Table 3 present the results of the ML analysis that discriminated between the subjects in Groups N and CV using pulse indices as features. Among the eight methods, LDA had the highest accuracy and AUC (area under receiver operating characteristic curve). The mean accuracy, specificity, sensitivity, and AUC of LDA were 69.77%, 0.54, 0.81, and 0.67, respectively, which was close to an acceptable discrimination level.

Table 4 lists the results of the ML analysis that discriminated between the subjects in Groups N and CV by using 23 clinical data as features. Among the eight methods, GNB had the highest accuracy and AUC. The mean accuracy, specificity, sensitivity, and AUC of GNB were 59.69%, 0.80, 0.45, and 0.63, respectively. The accuracy and AUC were lower than those in Table 3 when using pulse indices as features.

**Figure 7:**
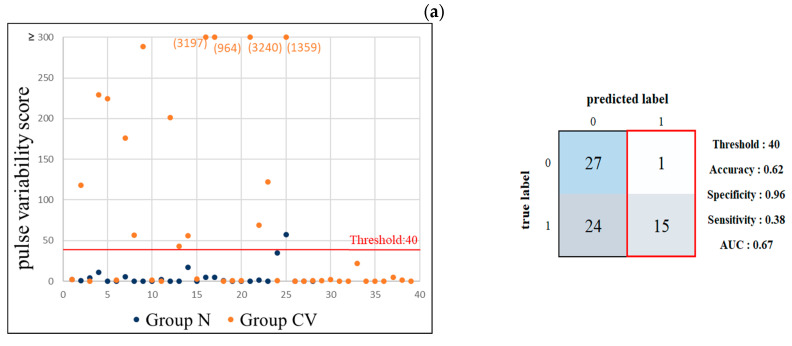

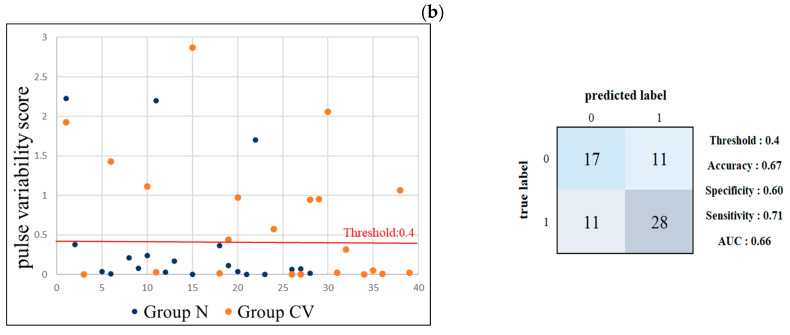
Results of pulse-variability score analysis of BPW indices for discrimination between Groups N (0; *n* = 28) and CV (1; *n* = 39). (**a**) Threshold = 40 produced accuracy = 62.68%, specificity = 0.96, sensitivity = 0.38, and AUC = 0.67. All but one of the subjects with a score >40 (*n* = 16; right side of the confusion matrix) experienced cardiovascular side effects. (**b**) Threshold = 0.4 produced accuracy = 67.16%, specificity = 0.60, sensitivity = 0.71, and AUC = 0.66. The sensitivity of 0.71 indicates that 71% of the subjects with cardiovascular side effects (the lower half of the confusion matrix) were accurately identified.

Figure 7 lists the performance results of using the self-developed pulse-variability scoring system to discriminate between the subjects in Groups N and CV. When using 40 as the threshold score, the mean accuracy, specificity, sensitivity, and AUC of LDA were 62.68%, 0.96, 0.38, and 0.67, respectively. All but 1 of the 16 subjects with a score >40 experienced cardiovascular side effects. When using 0.4 as the score threshold, the mean accuracy, specificity, sensitivity, and AUC of LDA were 67.16%, 0.60, 0.71, and 0.66, respectively.

**Figure 8:**
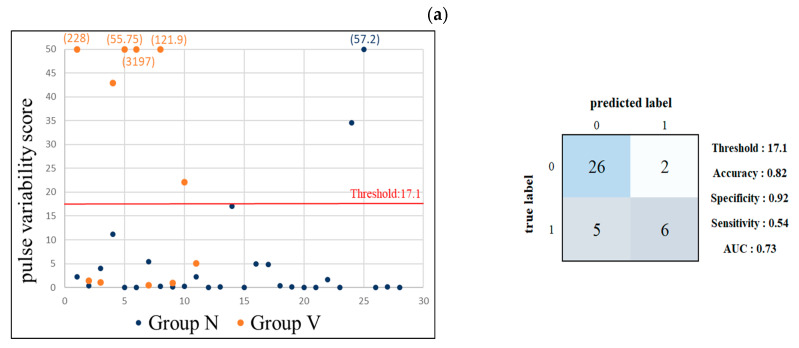

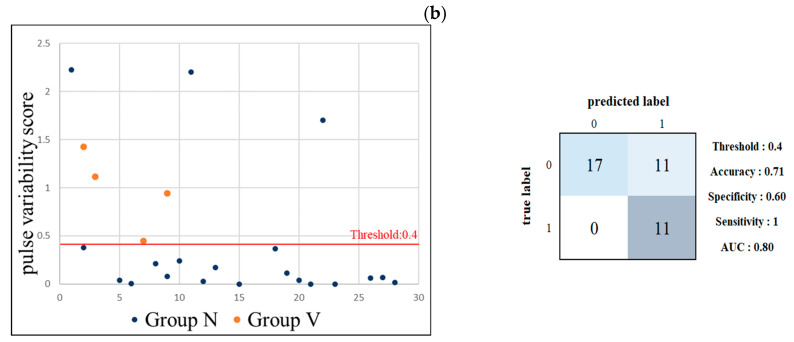
Results of pulse-variability score analysis of BPW indices for discrimination between Groups N (0; *n* = 28) and V (1; *n* = 11). (**a**) Threshold = 17.1 produced accuracy = 82.05%, specificity = 0.92, sensitivity = 0.54, and AUC = 0.73. This analysis achieved a very high specificity and an acceptable discrimination level. The AUC value was higher than that between Groups N and CV (Figure 6). (**b**) Threshold = 0.4 produced accuracy = 71.79%, specificity = 0.60, sensitivity = 1, and AUC = 0.80. The AUC of 0.80 indicates an excellent discrimination level. All of the subjects with vascular side effects were accurately identified (sensitivity = 1.00).

Figure 8 lists the performance results when using the pulse-variability score to discriminate between the subjects in Groups N and V. When using 17.1 as the score threshold, the mean accuracy, specificity, sensitivity, and AUC of LDA were 82.05%, 0.92, 0.54, and 0.73, respectively, which indicated an acceptable discrimination level. When using 0.4 as the score threshold, the mean accuracy, specificity, sensitivity, and AUC of LDA were 71.79%, 0.60, 1.00, and 0.80, respectively. The AUC value (which indicated an excellent discrimination level) was higher than that between Groups N and CV (Figure 7). All of the subjects with vascular side effects were accurately identified (sensitivity = 1).

**Figure 9:**
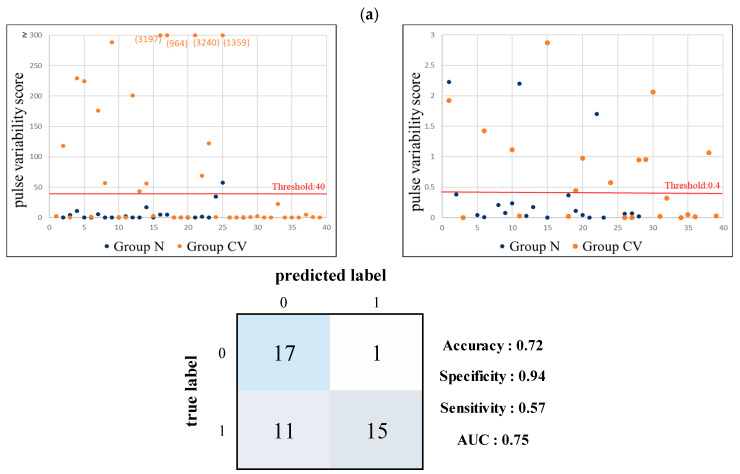

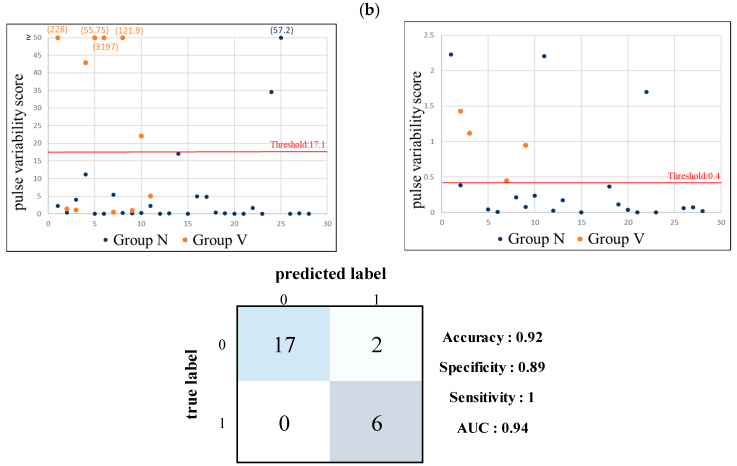
Results of pulse-variability score analysis of BPW indices for discrimination by excluding the data points located between the two thresholds. (**a**) Between Groups N (0; *n* = 28) and CV (1; *n* = 39). The two thresholds were 40 and 0.4. The excluded proportion was 34.33% (23/67). Accuracy = 72.72%, specificity = 0.57, sensitivity = 0.94, and AUC = 0.75. (**b**) Between Groups N (0; *n* = 28) and V (1; *n* = 11). The two thresholds were 17.1 and 0.4. The excluded proportion was around 35.90% (14/39). Accuracy = 92.00%, specificity = 1.00, sensitivity = 0.89, and AUC = 0.94.

We then changed the score range to exclude data around the borderline to avoid the interference of ambiguity. As shown in Figure 9, we used 0.4–40 as the threshold range between Groups CV and N. The proportion of the adopted data outside the ambiguity range was 65.7% (44/67); the accuracy, specificity, sensitivity, and AUC were 72.72%, 0.57, 0.94, and 0.75, respectively. When using 0.4–17.1 as the threshold range for Group V, the proportion of the adopted data was 64.1% (25/39); the accuracy, specificity, sensitivity, and AUC were 92.00%, 1.00, 0.89, and 0.94, respectively.

Table 5 indicates that there were no significant changes in HR and HR_CV in any of the four groups.

## 4. Discussion

This study found prominent changes in spectral BPW indices following BNT162b2 vaccination, especially in the pulse-variability indices. The ML and pulse-variability score analyses represent possible methods for discriminating between subjects with cardiovascular side effects.

### 4.1. Changes in the Pulse Indices

COVID-19 infection can affect different levels of the cardiovascular system [24]. Regarding the heart, myocardial injury is frequently observed in patients [25]. The suggested cardiac sequelae include heart failure, cardiomyopathy, acute coronary syndrome, and arrhythmia [8]. Regarding blood vessels, COVID-19 infection may result in immune-mediated damage to the systemic vasculature [8]. The stiffness of larger arteries is higher in patients with moderate and severe COVID-19 than in patients with mild COVID-19. The induced mechanical fatigability of the arterial wall can lead to increased ventricular afterload and impaired coronary perfusion [24]. COVID-19 infection is strongly associated with arterial and venous thrombus formation, and hence affects the vasculature impedance [8,10]. Regarding microcirculation, COVID-19 can infect the endothelial cells to severely alter the microcirculation and cause endothelial inflammation [10]. Regarding blood flow, using cutaneous local thermal hyperemia as a stimulus, the endothelium-dependent microvascular vasodilator response was found to be markedly decreased [9]. The mechanisms underlying this vascular damage may include endothelialitis and vascular thrombosis [10,26]. Vasculitis may contribute to thrombosis, hemodynamic instability, and autonomic dysregulation, which may all lead to impairments of vascular integrity and tissue inflammation [26]. The adverse cardiovascular events associated with mRNA COVID-19 vaccination range from inflammation to thrombosis and ischemia. Increased systemic reactogenicity and immunogenicity after vaccination have been reported in Pfizer-BioNTech clinical trials [13].

Figure 2 compares changes in the pulse indices (of BPW and PPG) before and after vaccination. Few significant changes were noted, illustrating that the BNT162b2 vaccination did not induce prominent effects on the cardiovascular function of all patients in the sample. This also implies similar effects on the pulse transmission condition both upstream (BPW) and downstream (PPG) of the artery.

In comparisons of pulse waveform indices *C_n_* and *P_n_* among the side effects groups, Figure 3 and Figure 4 indicate the changes were more prominent for BPW indices than for PPG indices; the subsequent discussion and ML and pulse-variability score analyses therefore focused on BPW indices. These results indicated that the only prominent effect on the pulse waveform indices was in the higher frequency components of Group V. Since the pulse can distend the arterial wall and push blood through the arteriolar tissue openings, *C_n_* can represent the power of each frequency component within the BPW. The main frequency components (lower frequency) that comprise a larger greater proportion of the BPW power were more closely related to the pulse transmission in the main artery, and the higher frequency components were more closely related to the pulse transmission in the peripheral arteries. Based on the above conjecture, it is possible that the increased higher frequency *C_n_* values were related to increased peripheral arterial stiffness in subjects with vascular side effects (Group V) following vaccination.

The main finding of the present pulse analysis was the changes in pulse-variability indices (*CV_n_* and *P_n_*_*SD*) following vaccination. Again, the most prominent changes were in the indices of Group V, with significant increases in all *P_n_*_*SD* indices. Cardiovascular variability indices have been widely studied to determine their possible relationships with cardiovascular regulatory function. For example, HR variability and BP variability indices have been used for monitoring changes in the regulatory activities induced by aging and disease [27,28]. For the vascular properties and blood flow, changes in the cardiovascular variability indices were also found to be correlated with the responses induced by disease or external stimulation [18,29]. For example, the pulse-variability indices were found to be higher in subjects with metabolic syndrome than in the controls, illustrating a stronger response to the changes in vascular stiffness and resistance induced by metabolic syndrome, that comprise a cluster of vascular risk factors [19]. mRNA COVID-19 vaccination has been suggested to be associated with adverse cardiovascular events such as thrombosis and ischemia [13]. In the present Group V, changes in these vascular properties may have caused challenges to the cardiovascular regulatory system to maintain blood supply homeostasis, and hence increased the instability of the pulse transmission condition. This may partly account for the increased values of pulse-variability indices noted in the present study.

The findings in Table 5 indicate that in all four groups, HR and HR_CV did not differ before and after vaccination. HR and HR_CV were correlated with the heartbeat and its regulatory activities; the present pulse-variability indices may be more strongly correlated with vascular condition. The present findings illustrate that even in the absence of a significant difference in HR and its variability index, the pulse-variability indices can still aid in the discrimination between cardiovascular side effects following vaccination.

Figure 5 compares changes in the BPW indices following vaccination between subjects with at least one (subGroup A) and without any one (subGroup B) of hypertension, hyperlipidemia, and hyperglycemia. Although non-significant, the most prominent differences can be noted in *P_n_*_*SD* indices in Group V. It is possible that subjects in subGroup A may have more severe vascular lesions. The arterial elastic properties may be different from those in subGroup B, and hence changes in the BPW indices following vaccination can be different between subGroups A and B.

### 4.2. ML Discrimination

The significant changes in some pulse indices hinted at using pulse indices as features in ML analysis, which may aid in the discrimination between cardiovascular side effects following BNT162b2 vaccination. In the ML analysis results between Groups CV and N (listed in Table 3), LDA had the highest AUC (0.67) among the eight models. This was higher than when using 23 clinical data as features, in which the AUC for GNB and SVM peaked at 0.63 (Table 4). The accuracy (around 60%) was also lower than when using pulse indices as features (around 70%). These findings illustrate the feasibility in discrimination between the cardiovascular side effects by using pulse indices as features.

In Table 3 (using pulse indices as features), LDA had the highest likelihood ratio of 1.76. In Table 4 (using 23 clinical parameters as features), GNB had the highest likelihood ratio of 2.25. This illustrated that using GNB and clinical parameters as features had a higher probability of correctly predicting disease in ratio to the probability of incorrectly predicting disease (indicated by a higher likelihood ratio value) than using LDA and pulse indices as features.

### 4.3. Pulse Variability Score Discrimination

Since only a small proportion of the present subjects had cardiovascular side effects, the sample size may not be adequate to form a reliable basis for ML analysis, although the application of cross-validation may have partially solved this problem. We therefore tried to develop a scoring system based on the present comparison of pulse index changes (as shown in Figure 3 and Figure 4) with the aim of providing an alternative method for discriminating between subjects with and without cardiovascular side effects. This may help to overcome the problem of the small sample.

According to Figure 3, the most prominent changes occurred in the pulse-variability indices of BPW, which we therefore selected as the features to construct the pulse-variability scoring system. As stated above, the changes in the pulse-variability indices can be related to the changes in the cardiovascular regulatory activities. Based on this method, factors that may change the pulse-variability score can be understood more easily, which is unlike the black-box condition implicit in an AI analysis.

Using the pulse-variability score analysis illustrated in Figure 7, when using either 40 or 0.4 as threshold, the AUCs for discriminating between Groups CV and N were close to those in the ML analysis. It is also worth noting that in the right half of the confusion matrix when using 40 as threshold, almost all of the subjects (15/16) with a score >40 had cardiovascular side effects. This suggests that a pulse-variability score of >40 can aid in the practical detection of cardiovascular side effects.

While the changes in pulse indices were the most prominent in Group V, the sample in this group was even smaller, and we therefore tried to use the pulse-variability score analysis to discriminate between the subjects with vascular side effects. Figure 8 indicates that using a threshold of 0.4 can achieve an excellent discrimination level. When using a threshold of 17.1, similar to the case in the right half of the confusion matrix when using a threshold of 40 in Group CV, most of those subjects (6/8) with a score >17.1 had vascular side effects. When using a threshold of 0.4, the specificity was 1.00, which indicated that all of the subjects with vascular side effects can be accurately detected. These findings illustrate that pulse-variability score analysis can aid in the practical detection of vascular side effects with high specificity and AUC.

As stated above, using different thresholds in the pulse-variability score analysis may have advantages and disadvantages, and combining two thresholds together may therefore enhance the discrimination advantages. Figure 9 indicates that using pulse-variability score analysis and excluding the data points located inside the two thresholds can further improve the AUC. The proportions that the adopted data points occupied were 65.7% and 64.1% for Groups CV and V, respectively. In practical application, these findings indicate that the pulse-variability score analysis cannot provide suggestions for only around one-third of subjects; for the rest (around two-thirds), the pulse-variability score analysis can reliably predict possible cardiovascular or vascular side effects that will follow vaccination.

## 5. Conclusions

The present findings and the related conclusions can be summarized as follows:

▪ Prominent effects were noted in Group V on the pulse harmonic indices induced by cardiovascular side effects following BNT162b2 vaccination.
▪ ML and pulse-variability score analyses can aid the discrimination between subjects with cardiovascular side effects. The score analysis can also provide information to aid the detection of the cardiovascular or vascular side effects.
▪ When excluding possible ambiguous data points (the adopted proportion was around two-thirds), the AUCs of the score analysis could be further improved to 0.94 and 0.75 for vascular and cardiovascular side effects, respectively.
▪ The present findings illustrate that combining the present noninvasive pulse measurement, frequency domain pulse waveform analysis, and the ML and pulse-variability score analyses can be a time-saving and easy-to-use method for detecting the changes in vascular properties associated with the cardiovascular side effects following BNT162b2 vaccination.
▪ The present results were mostly limited by the small proportion of subjects with cardiovascular side effects. Future studies should therefore focus on accumulating more data to reinforce the reliability of the ML and pulse-variability score analyses. The present technique can also be applied to study the effects of other COVID-19 vaccinations (e.g., Moderna and AZ vaccines) on vascular properties.

## Figures and Tables

**Table 1:** Characteristics of the study subjects in Groups N, CV, V, and C.

(a) BPW	Group N		Group CV		Group V		Group C	
	Male	Female	Male	Female	Male	Female	Male	Female
Subject number	17	11	29	10	7	4	25	9
	28		39		11		34	
Age	48.6 ± 11.7	40.6 ± 13.2	39.8 ± 17.7	31.6 ± 12.4	41.0 ± 16.0	35.0 ± 11.1	41.0 ± 4.2	31.3 ± 4.0
	45.5 ± 12.9		37.7 ± 16.9		38.8 ± 14.7		38.4 ± 4.2	
BMI	23.2 ± 3.7	24.2 ± 3.5	22.6 ± 4.1	24.3 ± 3.9	22.8 ± 2.9	24.4 ± 3.6	22.6 ± 4.2	24.6 ± 4.0
	23.6 ± 3.6		23.0 ± 4.1		23.4 ± 3.3		23.2 ± 4.2	
Hypertension	1	2	3	2	0	1	3	2
	3		5		1		5	
Hyperlipidemia	9	4	10	2	3	2	9	2
	13		12		5		11	
Hyperglycemia	1	0	2	3	0	2	2	2
	1		5		2		4	
**(b) PPG**	**Group N**		** Group CV**		** Group V**		** Group C**	
	** Male**	** Female**	** Male**	** Female**	** Male**	** Female**	** Male**	** Female**
Subject number	9	4	15	2	5	2	13	1
	13		17		7		14	
Age	45.7 ± 16.4	40.8 ± 13.2	45.1 ± 16.4	28.5 ± 5.5	51.4 ± 6.2	28.5 ± 5.5	44.8 ± 17.6	23.0
	44.2 ± 15. 7		43.1 ± 16.4		44.9 ± 11.9		43.3 ± 17.9	
BMI	22.7 ± 3.9	24.7 ± 2.2	23.0 ± 4.2	21.1 ± 0.8	23.9 ± 2.6	21.1 ± 0.8	22.7 ± 4.4	20.3
	23.3 ± 3.6		22.8 ± 4.0		23.1 ± 2. 6		22.6 ± 4.2	
Hypertension	0	0	2	0	0	0	2	0
	0		2		0		2	
Hyperlipidemia	5	2	7	1	2	1	7	0
	7		8		3		7	
Hyperglycemia	0	0	2	0	0	0	2	0
	0		2		0		2	

**Table 2:** Definitions of cardiac and vascular side effects accompanying COVID-19 vaccination.

	Item	Normal Range
Cardiac side effect	S3 (3rd heart sound)	≤5
	S4 (4th heart sound)	≤5
	EMAT (Electromechanical Activation Time)	≤120 (ms)
	SDI (Systolic Dysfunction Index)	≤5
	X-ray cardiothoracic ratio	<0.5
	Troponon-I	0–26.2 (pg/mL)
	NT-proBNP	<125 (pg/mL)
Vascular side effect	Creatinine	0.57–1.11 (mg/dL)
	D-Dimer	<0.5 (mg/L)

**Table 3:** Results of ML analysis (eight methods) for BPW indices in discriminating between Groups N and CV using pulse indices as features. Accuracies are presented as percentages. Threefold cross-validation values are listed. “*” denotes the highest average value. Among the eight methods, LDA had the highest accuracy and AUC (area under receiver operating characteristic curve).

	SVM	MLP	GNB	DT	RF	LR	LDA	KNN
Accuracy	59.40	59.71	60.29	52.41	59.33	62.66	69.77 *	57.11
AUC	0.55	0.58	0.57	0.52	0.58	0.60	0.67 *	0.55
Specificity	0.25	0.47	0.38	0.47	0.48	0.47	0.54 *	0.41
Sensitivity	0.84 *	0.69	0.76	0.56	0.68	0.73	0.81	0.68

**Table 4:** Results of ML analysis (eight methods) for BPW indices between Groups N and CV using clinical data (23 parameters) as features. Accuracies are presented as percentages. Threefold cross-validation values are listed. “*” denotes the highest average value. The 23 clinical data points included the following: uric acid, cholesterol, HDL, LDL, GOT, GPT, glucose (random), hs-CRP, WBC, RBC, HGB, HCT, MCV, MCH, MCHC, RDW-CV, platelets, neutrophils, lymphocytes, monocytes, eosinophils, basophils, and ESR. Among the eight methods, GNB had the highest accuracy and AUC (area under receiver operating characteristic curve), which were lower than those in Table 2, for which pulse indices were used as features.

	SVM	MLP	GNB	DT	RF	LR	LDA	KNN
Accuracy	59.69 *	48.18	59.69 *	55.00	48.18	53.18	43.33	40.00
AUC	0.50	0.43	0.63 *	0.51	0.44	0.46	0.41	0.37
Specificity	0.00	0.20	0.80 *	0.36	0.24	0.13	0.31	0.25
Sensitivity	1 *	0.67	0.45	0.67	0.64	0.80	0.51	0.50

**Table 5:** No significant changes in HR (in beats/minute) and HR_CV (in %) were found in any of the four groups.

Group	N	CV	V	C
Pre-HR	76.26 ± 9.88	82.97 ± 9.98	86.1 ± 8.08	83.09 ± 10.36
Post-HR	78.42 ± 9.95	83.71 ± 11.87	86.29 ± 8.55	83.59 ± 12.11
Pre-HR_CV	4.04 ± 1.96	4.46 ± 2.34	3.53 ± 1.66	4.48 ± 2.44
Post-HR_CV	3.89 ± 2.13	4.24 ± 2.12	3.94 ± 2.2	4.21 ± 2.16

## Data Availability

The data presented in this study are available on request from the corresponding author. The data are not publicly available due to ethical concern.

## Supplementary Materials

The following supporting information can be downloaded at: https://www.mdpi.com/article/10.3390/jcm11206119/s1, Table S1. Assessment schedule; Table S2. Laboratory examination; Table S3: 12-lead EKG; Table S4. Chest X-ray investigation; Table S5. Side-effect investigation of COVID vaccine.
