# Supplementary material for: Machine Learning Analyses Revealed Distinct Arterial Pulse Variability According to Side Effects of Pfizer-BioNTech COVID-19 Vaccine (BNT162b2)
Source: J Clin Med. 2022 Oct 17;11(20):6119. doi: 10.3390/jcm11206119 (PMC9605457; doi:10.3390/jcm11206119)
Supplement: Supplementary file 1 [file jcm-11-06119-s001.zip › jcm-1925815-supplementary.pdf]

S1. Measurements

Before the measurements, the subjects were relaxed and rested for at least 10 min. The environmental temperature was within 23-25 °C during the entire measuring period. All subjects gave their informed consent before experiments commenced, were asked to not take any medication for 3 days before experiments, and did not consume food at least 1 h before each experiment.

For each experiment, the subjects were sitting on a chair, and 1-minute BPW and PPG signals were measured noninvasively. The BPW signal was acquired by a pressure transducer (KFG-2-120-D1-11, Kyowa) held onto the skin surface above the radial artery 2 cm from the left wrist. The PPG signals from a 940-nm-wavelength infrared LED penetrating the middle finger tissue were acquired by photodiodes. The signals were connected to a self-made current-to-voltage converter circuit, and then connected to an analog-to-digital converter card (PCI- 9111DG, Adlink Technology, Taiwan) operating at a sampling rate of 1024 Hz. Before the measurement, the heart rate (HR), brachial systolic BP and diastolic BP were measured by using a sphygmomanometer (MG150f, Rossmax).

One thermistor was attached around the wrist to monitor the skin-surface temperature. The resistance of the thermistor was transformed into voltages (by a custom-made circuit) that were also sampled every minute by the analog-to-digital converter card. The acceptable range for the temperature stability during the baseline period was a temperature variation of less than 1.0 °C.

Table S1. Assessment schedule

	M0 (Pre-Vaccine)	M1 (Post-Vaccine)
	On the vaccine day	7days (±3days) after the vaccine
Inclusion/ Exclusion criteria	x	
Inform Concern Form	x	
Demography	x	
Vaccine Information	x	x
Body Height/ Weight	x	
Vital sign	x	x

BPW measurement	x	x
Laboratory examination	x	x
12-lead EKG	x	x
Chest X-ray	x	x
Side effect		x
Event assessment	x	x

Table S2. Laboratory examination

Laboratory examination				
1.WBC	WBC	2.DC	Neutrophil	7. Creatinine
	RBC		Lymphocyte	8. GOT
	HGB		Monocyte	9. GPT
	HCT		Eosinophil	10. Glucose(random)
	MCV		Basophil	11. Cholesterol
	MCH	3. ESR		12. LDL
	MCHC	4. hs-CRP		13. HDL
	RDW-CV	5. Troponin-I		14. NT-proBNP
	Platelet	6. Uric Acid		15. d-dimer

Table S3. 12-lead EKG

12-lead EKG	
Pre-EKG	Qtc
Vent rate	P axes
PR int	R axes
QRS dur	T axes
QT	ST elevation

Table S4. Chest X-ray investigation

Chest X-ray findings	
Pulmonary edema	Yes ☐ No ☐
Cardiothoracic ratio	

Table S5. Side-effect investigation of COVID vaccine

Side effect	
fever >38°C	
chills	Yes ☐ No ☐
fatigue	Yes ☐ No ☐

headache	Yes ☐ No ☐
myalgia	Yes ☐ No ☐
arthralgia	Yes ☐ No ☐
injection site pain	Yes ☐ No ☐
injection site lump	Yes ☐ No ☐
injection-side axillary tenderness or swelling	Yes ☐ No ☐
other	Yes ☐ No ☐
myocarditis	Yes ☐ No ☐

S2. Analysis

The present analysis procedure included signal processing and information processing:

- signal processing

Frequency-domain analysis was applied to derive the 40 harmonic indices from the measured BPW signal (n=1-10): amplitude proportion (C_n), coefficient of variation of C_n (CV_n), phase angle (P_n), and standard deviation of P_n (P_n_SD).

Each individual pulse (between foot points) can be represented by the following finite series. The pulses were excluded if the values between the two foot points were larger than 20% of the pulse amplitude.

$$x(t) = \frac{A_0}{2} + \left\{ \sum_{n=1}^{k/2} A_n \cos n\omega t_s + \sum_{n=1}^{k/2} B_n \cos n\omega t_s \right\}$$

The Fourier coefficients (A_n and B_n) of the pulse can be calculated as

$$A_n = \frac{2}{k} \sum_{s=0}^{k} x_s \cos n\omega t_s \;\; (for\; n = 0,1,..., \frac{k}{2})$$

$$B_n = \frac{2}{k} \sum_{s=0}^{k} x_s \sin n\omega t_s \;\; (for\; n = 0,1,..., \frac{k}{2})$$

where ω is the angular frequency and t_s is the sampling time interval.

The amplitude (Amp_n) and phase angle (P_n) of each harmonic of the pulse harmonic spectrum can then be calculated as $Amp_n = \sqrt{A_n^2 + B_n^2}$ and $P_n = \arctan(B_n / A_n)$. The amplitude proportions (C_n values) for each pulse were calculated as $Amp_n / Amp_0 \times 100\%$, for n = 1–10. CV_n was then

calculated as the coefficient of variations of C_n, and P_n_SD was calculated as the standard deviation of P_n.

Signal processing was performed with MATLAB (MathWorks). The differences in the fundamental physiological parameters were tested with t-test. The differences in pulse indices before and after the vaccination were tested with paired t-test and were considered significant when $p<0.05$; all p-values were two-sided hypotheses.

- information processing

For information processing, the features of pulse signals were collected from the results of the signal-processing stage described above, to yield 40 indices for each pulse: C_n, CV_n, P_n, and P_n_SD values for n = 1–10. Each feature was scaled by Z-score normalization to eliminate the effects of the variations in the ranges of different indices. Python (version 3.7) was used as the analysis tool in the information processing; eight machine-learning methods were used to classify the data (details of model parameters are listed in the following table).

Threefold cross validation was used in the model training stage. The proposed classification model was evaluated by calculating the accuracy, AUC (area under the receiver operating characteristics curve), sensitivity and specificity.

Parameters of the machine-learning models.

machine-learning methods	model parameters
SVM (support vector machine)	C=1; kernel: rbf; gamma: auto; tol= 0.0001; max_iter=-1; class_weight: none
MLP (multilayer perception)	hidden_layer_sizes=100; solver: adam; alpha=0.0001; batch_size: auto; max_iter=200; learning_rate_int=0.001
GNB (Gaussian Naive Bayes)	Priors: none
DT (decision tree)	Criterion: gini; Splitter: best; max_depth: none; min_samples_split=2; min_samples_leaf=1; min_weight_fraction_leaf=0; max_features: none; max_leaf_nodes: none; min_impurity_split=0.0
RF (random forest)	n_estimators=100; criterion: gini; max_depth: none; min_samples_split=2; min_samples_leaf=1; min_weight_fraction_leaf=0; max_features: none; max_leaf_nodes: none
LR	Penalty: l2; Solver: lbfgs; multi_class: auto; class_weight:

(logistic regression)	none
LDA<br>(linear discriminant analysis)	Solver: svd; Shrinkage: none; Priors: none
KNN<br>(K-nearest neighbor classification)	n_neighbors=5; weights: uniform; algorithm: auto; n_jobs: none; p: none
